# Validation of miRNA genes suitable as reference genes in qPCR analyses of miRNA gene expression in Atlantic salmon (*Salmo salar*)

**DOI:** 10.1186/1756-0500-7-945

**Published:** 2014-12-23

**Authors:** Ilona Johansen, Rune Andreassen

**Affiliations:** Faculty of Health Sciences, Oslo and Akershus University College of Applied Sciences, Oslo, Norway

## Abstract

**Background:**

MicroRNAs (miRNAs) are an abundant class of endogenous small RNA molecules that downregulate gene expression at the post-transcriptional level. They play important roles by regulating genes that control multiple biological processes, and recent years there has been an increased interest in studying miRNA genes and miRNA gene expression. The most common method applied to study gene expression of single genes is quantitative PCR (qPCR). However, before expression of mature miRNAs can be studied robust qPCR methods (miRNA-qPCR) must be developed. This includes identification and validation of suitable reference genes. We are particularly interested in Atlantic salmon (*Salmo salar*). This is an economically important aquaculture species, but no reference genes dedicated for use in miRNA-qPCR methods has been validated for this species. Our aim was, therefore, to identify suitable reference genes for miRNA-qPCR methods in *Salmo salar*.

**Results:**

We used a systematic approach where we utilized similar studies in other species, some biological criteria, results from deep sequencing of small RNAs and, finally, experimental validation of candidate reference genes by qPCR to identify the most suitable reference genes. Ssa-miR-25-3p was identified as most suitable single reference gene. The best combinations of two reference genes were ssa-miR-25-3p and ssa-miR-455-5p. These two genes were constitutively and stably expressed across many different tissues. Furthermore, infectious salmon anaemia did not seem to affect their expression levels. These genes were amplified with high specificity, good efficiency and the qPCR assays showed a good linearity when applying a simple cybergreen miRNA-PCR method using miRNA gene specific forward primers.

**Conclusions:**

We have identified suitable reference genes for miRNA-qPCR in Atlantic salmon. These results will greatly facilitate further studies on miRNA genes in this species. The reference genes identified are conserved genes that are identical in their mature sequence in many aquaculture species. Therefore, they may also be suitable as reference genes in other teleosts. Finally, the systematic approach used in our study successfully identified suitable reference genes, suggesting that this may be a useful strategy to apply in similar validation studies in other aquaculture species.

**Electronic supplementary material:**

The online version of this article (doi:10.1186/1756-0500-7-945) contains supplementary material, which is available to authorized users.

## Background

MicroRNAs (miRNAs) are an abundant class of endogenous small RNA molecules. They regulate gene expression as part of the miRNA-induced silencing complex (miRISC) at the post-transcriptional level by binding to the mRNA of target genes in a sequence specific manner [1, 2]. Most mature miRNAs are 20–22 nt in length, and while some miRNAs are highly conserved from species to species, other miRNAs seems to be species specific [3–5]. They are often expressed in a tissue-specific manner and play important roles in multiple biological processes by regulating genes that control developmental timing, growth, stem cell division and apoptosis [6–9]. Failure in miRNA expression or failure in target gene recognition may result in genetic disease. There are e.g. more than 160 diseases reported in the miR2Disease database that are associated with dysfunction of miRNA genes or miRNA/target gene-interaction [10]. Dysfunctional miRNA/target gene interaction may also contribute to development of cancer when miRNAs e.g. act as oncogenes [11].

The fundamental role of miRNA genes as major regulators of the expression of protein coding genes and their role as causative mediators of disease [10, 11] have led to an increase in studies investigating the association between disease and miRNA gene expression. In farmed animals the motivation for miRNA studies is not only focused on their impact on health and disease, but also includes searches for miRNA variation that affects economically interesting traits (e.g. the “texcell” mutation in sheep) [12]. However, before any interesting biological variation in miRNA gene expression can be unraveled robust methods for quantitation must be developed. Recent years studies in many economically interesting aquaculture species have resulted in identification and characterization of a large number of conserved as well as species specific miRNA genes and their corresponding mature miRNAs [13–20]. Further studies of biologically interesting miRNA genes in these species will ultimately include studies on variation of their miRNA expression levels. We are particularly interested in Atlantic salmon (*Salmo salar*) [13]. Validation of quantitative methods dedicated for analyses of miRNA expression levels in Atlantic salmon would greatly benefit research in this species.

Advances in microarray and sequencing technology (deep sequencing methods) the last decade have led to increased sensitivity in detection of gene expression differences. These high throughput methods may be used to analyse expression of thousands of miRNA genes in parallel [21] while the expression of single miRNA genes may be studied by use of qPCR methods (miRNA-qPCR) designed to detect one miRNA gene at a time. When expression of miRNA genes (or any other gene) are quantified by use of high throughput methods it is common practice to verify, and more accurately measure, any interesting expression differences disclosed by follow-up studies using qPCR methods [21]. Thus, the expression of almost all interesting miRNA genes would therefore eventually be analysed by qPCR.

qPCR methods, and all other methods that measure relative gene expression differences, depend on some strategy to correct for non-biological sample to sample variation to be able to measure the true biological expression levels of the gene(s) of interest (target genes) in a set of samples. The usual approach to control for the effects of systematic experimental bias and technical variation is to utilize within sample measurements of other genes that are expressed in a constitutive and stable manner, so-called normalization genes or reference genes. Assuming that these reference genes are close to identically expressed in all samples, the differences in expression level of the target genes that are not caused by biological variation may be controlled by use of these endogenous references [22, 23]. Consequently, any qPCR method measuring relative expression differences can never have a better sensitivity or precision than that allowed from the expression level and biological variation of the reference genes. If using genes that are biased as reference genes this may in worst case lead to erroneous conclusions [24]. A proper normalisation gene show small non-biased biological variation across the groups of samples compared, the smaller the variation of the normalisation genes, the better the precision of the method. It is also common belief that any normalisation gene should be biologically similar to the target gene. This may be very important in quantitative measurements of miRNA gene expression since mature miRNAs are very short RNA molecules. Therefore, they may differ substantially from other RNA molecules traditionally used as endogenous controls (e.g. snRNAs) in important pre-processing parameters like RNA extraction efficiency, reverse transcription efficiency and degradation level. If the endogenous control is differently affected by sample pre-processing and differ in degradation level this may introduce unwanted technical variation and bias, especially in comparisons involving multiple tissues [25–27]. Considering the optimal structural features characterizing the ideal reference genes, the most suitable reference genes for any qPCR method measuring the expression of a particular miRNA gene would simply be another miRNA gene [22, 25, 28]. Furthermore, the ideal reference miRNA genes would be the ones that are constitutively expressed with minimal expression level differences between different tissues and states (e.g. normal vs. disease).

Several other studies have suggested particular miRNA genes that may be suitable as normalization controls in miRNA-qPCR methods. These reference genes have been identified by analyzing sample material from humans and different model species (e.g. mouse or *C. elegans*) when investigating expression levels across normal tissues or in certain disease states within some particular tissues (e.g. cancer) [22, 28, 29]. There is a lack of such validation studies among aquaculture species. The aim of this study was to identify suitable miRNA reference genes to be used as normalization controls in miRNA-qPCR methods in Atlantic salmon since we have a particular interest in this species. However, mature miRNA sequences as well as their functions are highly conserved. Therefore, such validation studies may also benefit research in other teleosts of economical importance to the aquaculture industry.

We have used a systematic approach where we utilize similar studies in other species, some biological criteria, results from deep sequencing of small RNAs in different tissues and, finally, experimental validation of a set of candidate reference genes by qPCR to identify the most suitable reference genes in Atlantic salmon. Validation studies from other species were first used to select a set of conserved miRNAs that were reported as constitutively expressed [22, 26, 29–31], assuming that a subset of the *Salmo salar* orthologs of these miRNA genes would show similar high stability in Atlantic salmon. Then some biological properties of the *Salmo salar* miRNA orthologs were considered and those that seemed as less good choices were removed. The remaining miRNA genes that still seemed to be good choices as reference genes in *Salmo salar* were compared regarding their stability in fifteen tissue samples retrieved from deep sequencing analysis of small RNA libraries. A subset of the miRNA genes, those that showed least variation across the deep sequencing samples, where finally validated by testing their performance in miRNA gene specific qPCR assays followed by identification of most suitable reference genes among the candidates by use of the NormFinder algorithm. These best performing reference genes were also tested in a panel of normal kidney samples vs kidneys samples from individuals with infectious salmon anaemia (ISA) to assure that the selected reference genes were not affected by general immune responses associated with virus disease.

## Results

### Selection of candidate miRNA reference genes

A total of 40 conserved miRNAs suggested as reference miRNA genes for qPCR from various validation studies were initially included as putative reference genes (most of them listed in Meyer et al.) [22, 26, 29–31]. To be applied as reference genes they must, however, be present in Atlantic salmon (have a miRNA ortholog in *Salmo salar*). Thus, the initially selected set of genes was first compared to the known miRNA genes in Atlantic salmon [13, 32]. This first comparison showed that, even if conserved in mammals, eight of the initially selected miRNA genes (miR-105, miR-191, miR-213, miR-324, miR-339, miR-374, miR-423 and miR-425) do not seem to have any orthologs in *Salmo salar*. These were, obviously, not further considered. An overview of the remaining candidate reference genes that were selected for our validation study (those with orthologs in Atlantic salmon) is shown in Table 1.

**Table 1:** **Summary of candidate genes selected for validation as reference genes**

miRNA gene ^1^	Source ^2^	Similar paralog ^3^	Clustered genes ^4^	CV Deep seq. ^5^	qPCR validation ^6^
ssa-miR-103-3p	Liang, P&L, Bar	no	not clustered	99,3	not included
ssa-miR-106a-5p	P&L	yes	25,93	-	-
ssa-miR-106b-5p	Liang	yes	25,93	-	-
ssa-miR-107-3p	Bar	no	not clustered	85,5	yes
ssa-miR139-5p	Bar	no	not clustered	138,5	not included
ssa-miR-140-3p	Liang	no	not clustered	172,6 (71,7)	yes
ssa-miR-148a-3p	Bar	yes	not clustered	-	-
ssa-miR-152-3p	Liang	no	not clustered	122,4	not included
ssa-miR-15b-5p	Liang	yes	16	-	-
ssa-miR-16a-5p	Liang, P&L, Appl	yes	15	-	-
ssa-miR-17-5p	P&L	no	18,19,20,92	77	yes
ssa-miR-183-5p	Bar	no	96,182	286,3	yes
ssa-miR-18a-5p	Bar	yes	17,19,20,92	-	-
ssa-miR-214-3p	Bar	no	not clustered	82,6	failed
ssa-miR-23a-3p	Bar	yes	24,27	-	-
ssa-miR-23b-3p	Bar	yes	24,27	-	-
ssa-miR-24a-3p P&L	yes	23,27	-	-	
ssa-miR-25-3p P&L	no	93,106	53,6	yes	
ssa-miR-26a-5p	Bar	yes	181	-	-
ssa-miR-26b-5p	Bar, Appl	yes	181	-	-
ssa-miR-29a-3p	Liang	yes	29b	-	-
ssa-miR-29b-3p	Liang	yes	29a	-	-
ssa-miR-30b-5p	Bar	yes	30e	-	-
ssa-miR-30d-5p	Bar	yes	30a	-	-
ssa-miR-30e-5p	Liang	yes	30d	-	-
ssa-miR-301a-3p	Genov	yes	not	clustered	-
ssa-miR-92a-3p	Liang, Bar, Appl	yes	17,18,19,20	-	-
ssa-miR-93a-5p	Liang, P&L, Bar	no	25,106	73,5	yes
ssa-miR-99-5p P&L	no	let-7c,7b	127,6	not	included
ssa-let-7a-5p P&L	yes	7f,100	-	-	
ssa-miR-455-5p	deep-seq	no	not clustered	128 (48)	yes
ssa-miR-210-5p	deep-seq	no	not clustered	184,9 (89,9)	yes

^1^All salmon miRNA genes selected for validation as reference genes annotated as in Andreassen et al. [13].

^2^The source for selecting each miRNA gene for validation is given in this column. References are Liang [30], P&L (Peltier and Latham) [26], Bar (Bargaje et al.) [31], Genov (Genovesi et al. [29]), Appl; Applied biosystems TaqMan controls, Deep-seq; these were suggested as reference genes by their high stability in deep sequence data.

^3^miRNA genes that has highly similar paralogous mature miRNAs in Atlantic salmon.

^4^If located in a miRNA gene cluster the identity of the other miRNA genes in the cluster is gives as their gene number.

^5^The covariation of all candidate miRNA genes that were selected for stability testing in deep-sequencing data. Covariation if removing one deep sequencing sample in three of the miRNAs (see Additional file 1) in parenthesis.

^6^Denoted yes means they were selected for experimental validation by qPCR.

An important property of any reference gene would be that it may be analysed with a high degree of specificity. The most common miRNA-qPCR methods utilize the mature miRNA sequence to achieve specific amplification of a certain miRNA gene, e.g. the miScript-assays used in our study where the specific amplification of a certain mature miRNA entirely depends on a single forward primer identical in sequence to the mature miRNA. However, a comparison of the mature sequences of the initially selected reference genes to all mature conserved miRNAs discovered in *Salmo salar* [13] revealed that there existed highly similar mature paralogous sequences to several of the candidate reference genes (e.g. so-called miRNA gene family members usually annotated -a, -b etc.). The amount of such paralogs seems to be larger in *Salmo salar* than other teleosts [13]. This may be explained by a fairly recent genome duplication in salmonids. Such mature sequences often differ only by a single nucleotide to the other family members and designing primers that are specific to one such mature miRNA would be challenging. The candidate genes that were members of such highly similar paralogous gene families were therefore considered less suitable as reference genes. An overview of the candidate genes that has mature sequences that are highly similar to other paralogous miRNA genes is given in Table 1. These miRNA genes were not included in further tests to be sure that any gene chosen as endogenous control would, independent of qPCR method used, be amplified with high specificity. Applying this criteria for selecting suitable reference genes from previous validation studies reduced the number of putative candidates to eleven miRNA genes (ssa-miR-103-3p, ssa-miR-107-3p, ssa-miR-139-5p, ssa-miR-140-3p, ssa-miR-152-3p, ssa-miR-17-5p, ssa-miR-183-5p, ssa-miR-214-3p, ssa-miR-25-3p, ssa-miR-93a-5p and ssa-miR-99-5p).

The location of the candidate miRNA genes in the Atlantic salmon genome was also considered as an important biological selection criterion. If two, or more, miRNA genes are located in a gene cluster they are often not expressed independently. Such clustered miRNA genes are most often co-transcribed, they respond and regulate similar gene pathways, and, as a consequence of this, the expression of one parallels the other [30]. Therefore, when using two reference miRNA genes they should not be clustered (transcribed from one “miRNA gene haplotype”) to avoid the possibility that the combined adjustment value is from a gene-pair with expression levels that mirror each other. Assessing the genome location of the reference candidates revealed that several genes were clustered (Table 1). Among the salmon miRNA genes with no paralogs there were only two miRNA genes, ssa-miR-93a-5p and ssa-miR-25-3p, that were located in a gene cluster in *Salmo salar* [13]. Thus, even if further tests showed that these two miRNA genes have a high stability, their location within the salmon genome suggests that only one of these should be chosen when using combined values from two miRNA genes for normalisation in a qPCR assay.

### Analysis of candidate reference genes stability in deep sequencing data sets

Candidate miRNA genes were compared regarding their stability across nine different tissues by use of deep sequencing data sets from a total of fifteen samples including the twelve samples used in a miRNA gene identification study in Atlantic salmon (Andreassen et al. [13]). The total miRNA read numbers of each sample was used to normalise the individual mature miRNA read numbers to retrieve expression values from each candidate miRNA gene that could be compared across samples (see Methods and Additional file 1). The relative standard deviation (coefficient of variation, CV) was then estimated for each of the eleven miRNA candidates. Finally, the CV’s were used to compare the individual stability of the eleven miRNAs (an approach similar to the one used in Bargaje et al. [31]). The datasets were also screened to identify other miRNA genes with high stability (low CV) to putatively discover new miRNA genes not suggested from other validation studies, but with properties that could make them suitable as reference genes in Atlantic salmon. Two more candidate genes (ssa-miR-455-5p and ssa-miR-210-5p) were included based on this screening of miRNA gene stability in the deep sequencing data sets (genes listed at the bottom in Table 1). The two new candidate miRNA genes both revealed a high degree of stability as well as passed the biological criteria we used for including reference genes (described in first section of results).

The coefficient of variation (CV) of each of the eleven candidate genes, as well as the CV of the two new miRNA genes added (ssa-miR-455-5p and ssa-miR-210-5p), is given in the column titled CV Deep seq. in Table 1. The comparison of the individual CV’s showed that there was five miRNA genes which seemed to be expressed in a more stable and constitutive manner than the others across all tissues. These were ssa-miR-25-3p, ssa-miR-17-5p, ssa-miR-93a-5p, ssa-miR-107-3p and ssa-miR-214-3p with CV’s ranging from about 53 to 86. These were all selected for further validation by qPCR analysis. Three of the other miRNAs (ssa-miR-140-3p, ssa-miR-455-5p and ssa-miR-210-5p) showed somewhat larger CV’s due to a difference in their expression level in one or two samples only (see Additional file 1). The CV’s of these miRNA genes when removing the samples showing a larger deviation in expression is given in parenthesis in Table 1. These three genes did appear as constitutively expressed at similar levels in all other samples. They were therefore, despite their somewhat larger deviation in one or two samples, included in the experimental validation by qPCR analysis. The largest relative variations across samples were in the five miRNA genes ssa-miR-103-3p, ssa-miR-139-5p, ssa-miR-152-3p, ssa-miR-99-5p and ssa-miR-183-5p. These appeared to be the least suitable reference genes, and among all genes compared ssa-miR-183-5p was the single one with the largest CV (286,3). To test whether there was a good correlation between the experimental validation by qPCR and the analysis of deep sequencing results this miRNA gene was, despite its lack of stability, also included for qPCR together with the eight other best performing ones. The four remaining candidate miRNA genes that revealed relatively large CV’s in our deep sequencing datasets (ssa-miR-103-3p, ssa-miR-139-5p, ssa-miR-152-3p and ssa-miR-99-5p) were not further validated. An overview of normalised read numbers and measurements of CV in the deep sequencing data is also given in Additional file 1.

### qPCR assay validation and stability testing by NormFinder

The miScript Cybergreen qPCR assay and custom designed forward primers identical to the mature-miRNA sequences of each of the nine miRNA genes (see Methods and Additional file 2) was used for qPCR analysis of mature miRNA expression levels. Melting point analysis of the qPCR products from each of the nine assays indicated that all primers were specific except the one used in the assay amplifying ssa-miR-214-3p. This assay showed poor and non-specific amplification (data not shown). Dilution series were used to test the linearity and efficiency of the eight other assays. All assays showed Pearson correlation coefficient values above 99.5 and with efficiencies between 94.5% and 108.1% (Additional file 2). This indicated that the miRNA-qPCR assays designed for analysing the candidate genes in general worked well. One assay, for analysing ssa-miR-214-3p, failed with the forward primer chosen. A closer inspection of the ssa-miR-214-3p mature sequences in *Salmo salar* (Additional file 1 in Andreassen et al. [13]) revealed that there exists two ssa-miR-214-3p mature sequences that are identical except they differ in length by one nucleotide at the 5’ end (5’acagcaggcacagacaggcaga 3’ and 5’uacagcaggcacagacaggcaga 3’). Similar length variation has been reported for other mature miRNAs e.g. miR-92 and miR-92 N in Liang et al. (personal communication [30]). We suspect that this is the reason why the ssa-miR-214-3p assay did not show specific amplification. Designing primers that could detect only one variant with high specificity would be challenging, and, in our opinion, the use of a miRNA gene with such a length variation as reference gene would be unwise. Therefore, no further attempts to optimize primers for this miRNA gene was carried out, and it was excluded from the group of putative reference genes. The remaining eight miRNA genes were relatively abundant, all showing relative expression values from medium to high when compared to the expression levels of other salmon miRNA genes (Additional file 2), and with average Ct-values that ranged between 18 and 28 when using template input as recommended by the manufacturer.

The eight candidate reference genes (ssa-miR-25-3p, ssa-miR-93a-5p, ssa-miR-17-5p, ssa-miR-455-5p, ssa-miR107-3p, ssa-miR-183-5p, ssa-miR-140-3p and ssa-miR-210-5p) were analyzed in four samples from each of seven different tissues; liver, kidney, brain, intestine, heart, white muscle and gills. The log Ct-values from all measurements (four samples in each of seven tissues) were transformed to linear values and used as input in NormFinder [33]. NormFinder is a data normalization tool that ranks the expression stability of candidate reference genes in a given sample set tested. The sample set tested in our analysis were grouped by different tissues (seven groups) so that the stability of the eight miRNA genes across different tissues were ranked by NormFinder. The NormFinder algorithm calculates a “stability value” that is inversely correlated with the stability of gene expression (so a higher stability value indicates lower stability). This ranking of each gene by a single stability value showed that six genes performed better than the others with stability values less than one (Table 2). The best single gene was ssa-miR-25-3p with a stability value of 0.462 while ssa-miR-93a-5p and ssa-miR-17-5p were the second and third best single genes (0.470 and 0.577, respectively). The best two-gene combination was, however, ssa-miR-107-3p and ssa-miR-455-5p (combined stability value 0.343).

**Table 2:** **Ranking of eight candidate reference miRNA genes based on average stability values across seven tissue groups as calculated by NormFinder**

miRNA gene	Stability value	Best combination of two genes	Stability value for best two-combination
ssa-miR-25-3p^1^	0,462	miR-107 and miR-455	0,343
ssa-miR-183-5p	1,443		
ssa-miR-140-3p	0,662		
ssa-miR-93a-5p	0,470		
ssa-miR-107-3p	0,841		
ssa-miR-455-5p	0,670		
ssa-miR-210-5p	1,133		
ssa-miR-17-5p	0,577		

^1^Best single miRNA gene.

We have a particular interest in studying miRNA genes and their role in virus disease. Therefore, to ensure that the expression level of any miRNA gene chosen as reference genes are not affected by any general response to virus disease, we investigated the effect of virus infection on the stability of the six best performing reference miRNA genes (stability values < 1, Table 2). Five kidney samples from healthy individuals and individuals with infectious salmon anemia (ISA), respectively, were used to compare whether any of the miRNA genes were differently expressed in normal vs ISA infected kidney. All six miRNA genes were analysed by miRNA-qPCR and ssa-miR-25-3p was applied as normalization gene (best performing single reference gene according to initial NormFinder analysis) to provide normalised Ct-values that could be used for comparison of the stability of the five other putative reference genes. The mean and standard deviations (SD) of the normalized Ct-values from each group is given in Additional file 3. These comparisons showed there were very similar expression levels in the two groups of ssa-miR-140-3p, ssa-miR-93a-5p, ssa-miR-455-5p and ssa-miR-17-5p. This indicated that ISA did not lead to any obvious expression changes in either of these miRNA genes. All expression values were normalized by use of Ct-values from ssa-miR-25-3p. These results did, therefore, also show that ssa-miR-25-3p was not affected by ISA. If ssa-miR-25-3p had been differently expressed in the ISA group, all values would be affected when normalized by this gene, and there would have been a difference between the two groups for any miRNA gene compared. However, they all showed similar expression levels in the two groups.

**Table 3:** **Ranking of five candidate reference miRNA genes based on average stability values across seven tissue groups as calculated by NormFinder**

miRNA gene	Stability value	Best combination of two genes	Stability value for best two-combination
ssa-miR_25-3p^1^	0,394	miR-25 and miR-455	0,337
ssa-miR_140-3p	0,830		
ssa-miR_93a-5p	0,449		
ssa-miR_455-5p	0,690		
ssa-miR-17-5p	0,431		

^1^Best single miRNA gene.

The comparison of the normalized expression level of ssa-miR-107-3p in the two groups did, however, show a larger difference in expression levels. Normal kidney tissues showed a mean normalized value of 1.42 with a SD of 0.34 while the ISA infected kidney tissues showed a mean normalized value of 3.47 with a SD of 0.7. A simple *t*-test showed that this difference was significant (p = 0.0002). We concluded that while expression of the five other miRNAs tested did not seem to be affected by ISA, the analyses of ssa-miR-107-3p indicated that this gene did change expression levels. Consequently, this miRNA gene would not be suitable as a reference gene when studying virus disease and miRNA expression.

**Figure 1:**
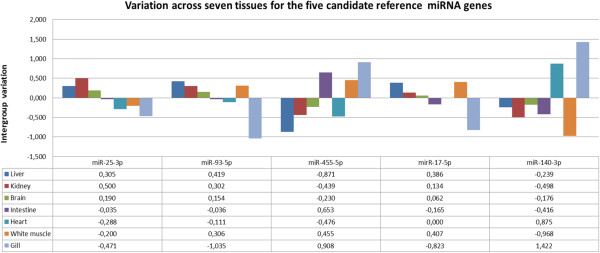
**The figure shows the variation across seven tissues of each of the candidate reference miRNA as calculated by the NormFinder algorithm.** The bar charts in the upper part of the figure illustrate the variation for each miRNA gene across tissues, while the corresponding inter-tissue stability values are given in the tables below the charts.

Our aim was to identify a small set of reference genes that may be applied in studies of different tissues and different conditions that included studies of virus disease. We therefore removed ssa-miR-107-3p from our group of suitable reference genes and performed a new NormFinder analysis using the remaining five best reference genes with stability values less than 1 from our initial test (ssa-miR-25-3p, ssa-miR-93a-5p, ssa-miR-17-5p, ssa-miR-455-5p and ssa-miR-140-3p). In addition the sample numbers were increased to eight in each tissue group to make the analysis more robust. The best single and best two gene combination as well as a ranking of the five genes by their stability values is given in Table 3. Also, Figure 1 illustrates the inter-tissue variation across all seven groups for the five miRNA genes analyzed. A manual inspection of the plots and stability values indicated that there was a fairly good correlation between the results from NormFinder analysis and the CV’s based on results from deep sequencing. The three miRNA genes ssa-miR-25-3p, ssa-miR-93a-5p and ssa-miR-17-5p were e.g. the ones with smallest CV’s (Table 1). These were also the ones showing the smallest stability values across the tissues tested by qPCR (Table 3 and Figure 1). Ssa-miR-183-5p was, on the other hand, the one with largest inter-tissue variation in both approaches applied (Table 2).

The best performing single gene was ssa-miR-25-3p (stability value 0.394) while the best performing two-gene combination was ssa-miR-25-3p and ssa-miR-455-5p (stability value 0.337). These genes, either ssa-miR-25-3p as single reference genes or in a two-gene combination with ssa-miR-455-5p, were, therefore, the most suitable reference genes for miRNA-qPCR across the conditions tested in our study.

## Discussion and conclusion

Our systematic approach led to the identification of reference genes suitable for miRNA expression studies in Atlantic salmon. The majority of the candidate miRNA genes included were initially selected because previous validation studies in vertebrates suggested they were suitable as endogenous controls. However, several of these did not seem to be suitable as reference genes in *Salmo salar*. The salmon ortholog of the candidate gene miRNA-183 showed e.g. relatively large amount of variation across tissues while the salmon ortholog of miRNA-107 was e.g. differently expressed in virus infected tissues. Results from Sarma et al. [34] also indicates that this miRNA gene is affected by virus disease in vertebrates. These findings support the practice that reference genes should be validated properly before being used in new species or when investigating particular conditions.

We used results from deep sequencing of several tissue samples to screen which candidate genes may be the more stable ones. Similar approaches have been applied in other validation studies [31]. In addition to test stability of pre-selected miRNAs the deep sequencing data may also be utilized for discovery of new potentially well-performing miRNA reference genes. This was also the case in our study where ssa-miR-455-5p was identified by screening deep sequencing data for stable miRNA genes. There was, in general, a good correlation between the measurements of miRNA gene stability in the deep sequencing datasets and those from the qPCR validation. Thus, to utilize deep sequencing data to identify miRNAs exhibiting a high degree of stability appears to be a useful approach to identify candidate miRNA genes to select for individual validation by qPCR.

In conclusion, we have identified two suitable reference genes for studying miRNA gene expression by qPCR in Atlantic salmon. Ssa-miR-25-3p was the best performing gene and together with ssa-miR-455-5p the best performing two-gene combination. They seem to be suitable as reference genes when studying miRNA expression across normal tissues in Atlantic salmon. They also performed well, with no obvious change in expression, in virus infected tissue. The two reference genes are conserved in vertebrates, even identical in their mature sequence in many aquaculture species, and the qPCR assays (miR-25-3p and miR-455-5p) provides good results without any change in primer sequences in rainbow trout and cod (data not shown). Although these genes need to be tested for their stability it is likely that they may be suitable as reference genes in other teleost species as well. The systematic approach used in our study seems to work well for identification of proper reference genes and may be applied in similar validations of reference genes in other aquaculture species.

## Material and methods

### Material

Two pre-smolt Atlantic salmon (*Salmo salar*) individuals, described in Andreassen et al. [13], were used for analysing miRNA expression by deep sequencing. Twelve of the deep sequencing samples were from this study [13], while three additional samples (kidney, head kidney and intestine, also from same individuals) were pre-processed and sequenced in the same manner as in [13]. The deep sequencing sample material, thus, included a total of 15 samples that were used for stability analysis in this study. Eight additional pre-smolt individuals were dissected and tissue samples from seven different organs were collected for isolation of total RNA. Following tissues were included; liver, kidney, heart, brain, gills, white muscle and intestine. These sample materials were used for the validation of the miRNA-qPCR method and the following stability testing from qPCR analyses of candidate reference genes (eight samples from seven normal tissues). Five kidney samples from individuals diagnosed with infectious salmon anaemia (ISA) were included to test whether reference gene expression were affected by virus disease. Dissection of fish and sampling of materials was performed by a certified veterinarian and in agreement with the provisions enforced by the Norwegian Animal Research Authority.

### Methods

#### Small RNA isolation

Total RNA was isolated from all samples by use of the mirVana miRNA isolation kit (Ambion) following the manufacturer’s protocol. RNA concentration and purity were determined using Nanodrop following the manufacturer’s protocol. The concentration of total RNA ranged from 40–900 ng/μl (total volume 100 μl).

#### cDNA synthesis and miRNA qPCR

The miScript assays were used for cDNA synthesis and qPCR as described by the manufacturer (Qiagen). A universal primer (reverse primer), provided with the miScript qPCR kit, was used in combination with our own custom designed forward primer in the qPCR amplification of each of the mature miRNA genes. The forward primers were designed based on the mature sequence of each of the miRNA genes to be amplified [13]. All primers were purchased from Sigma Aldridge, purified by desalt only and provided as liquid solution of 100 μM from the manufacturer. They were diluted to 10 μM for use in each of the qPCR assays, except the miR-17-3p assay and the 140-3p assay were 5 and 8 μM was used, respectively. All forward primer sequences are given in Additional file 2. The qPCR (quantitative PCR) was run on a Mx3000p (Stratagene). The qPCR reaction mixture consisted of 12,5 μL 2xQuantitec Syber Green Master Mix, 2,5 μL10x miScript Universal Primer, 2,5 μL of 10 μM forward miRNA gene specific primer, 5 μL Rnase free water, and 2,5 μL cDNA (miScript RT-PCR products diluted 1:10 prior to qPCR). Amplification was performed in 96-well plates. The following program was used: one thermal cycle at 95°C for 15 min followed by 40 cycles of 94°C for 15 sec, 55°C for 30 sec and 70°C for 30 sec. The Mx3000p software package (the cybergreen assay module) was used for qPCR analysis. The cybergreen assay module includes a final melting point analysis that follows the 40 cycles of quantitative PCR. Plots from melting point analysis were manually inspected for all miRNA gene assays tested to verify that forward primers were specific (data not shown). Tenfold dilution series of templates was analysed using the Mx3000p software to provide measurements of the linearity and efficiency of the individual assays (Additional file 2).

The effect of ILA virus infection on the stability of six selected miRNA genes (ssa-miR-25-3p, ssa-miR-93a-5p, ssa-miR-107-3p, ssa-miR-17-5p and ssa-miR-140-3p and ssa-miR-455-5p) was tested in the following manner. Five kidney samples from individuals infected with ILA and five kidney samples from normal individuals were analyzed by qPCR. The results (Ct-values) were normalized using ssa-miR-25-3p as normalization gene (best performing single reference gene). The normalized Ct-values from the five other miRNA genes were used to calculate the mean and standard deviations of their expression level in the normal kidney tissue group and in the ISA kidney tissue group. Comparisons of the mean and the spread of the five miRNA gene’s expression level in the two groups were used to reveal whether any gene was differently expressed. One miRNA (ssa-miR-107-3p) seemed to be differently expressed. A *t*-test was therefore performed to test whether the difference between expression in normal kidney tissue and ISA infected kidney tissues was significant (http://studentsttest.com/).

#### Stability testing of candidate reference genes

Deep sequencing data from 15 samples (see Additional file 1) was used to test stability of the candidate reference genes across nine different tissues in the following manner. Total number of miRNA reads from a sample (all reads perfectly matching a mature salmon miRNA) was retrieved by use of novoalign (http://www.novocraft.com). The total read numbers were used to normalize the read numbers of the individual mature miRNAs within each sample. The average normalised read numbers of each miRNA gene in the fifteen samples was then estimated along with their standard deviations. Finally, the relative standard deviation (coefficient of variation, CV) was estimated by dividing the mean normalised read number from each miRNA gene by their standard deviation. The CV was then used as a measurement of relative miRNA gene stability across the tissues included in the deep sequencing.

The data normalization tool NormFinder (http://moma.dk/normfinder-software) [33] was used to measure the stability of candidate genes. First, eight candidate miRNA genes were tested in seven different tissues (n = 4) and the results from each miRNA gene were grouped by tissues. Then the five best performing genes were tested in a new NormFinder analysis with input from measurements of eight samples in each of the seven tissue groups. All raw Ct values from measurements by qPCR were transformed to linear scale before used as input in NormFinder. The NormFinder algorithm calculates a “stability value” that is inversely correlated with the stability of gene expression (so a higher stability value indicates lower stability). This data normalization tool was, thus, used to rank the candidate genes by their stability and to identify most optimal two gene combination for normalization.

## Electronic supplementary material

Additional file 1: **Contains complete dataset of read numbers, normalised read numbers and CV’s of reference genes analysed in different tissue samples from deep sequencing.** (XLSX 15 KB)

Additional file 2: **Contains primer sequences of all forward primers and other parameters relevant to the miRNA-qPCR method applied.** (XLSX 12 KB)

Additional file 3: **Shows comparisons of expression levels of the candidate reference genes in normal vs ISA infected kidney tissue.** (XLSX 11 KB)
